# A case of ruptured aneurysm of coronary-pulmonary artery fistula diagnosed after emergency thoracotomy

**DOI:** 10.1186/s40792-018-0436-1

**Published:** 2018-03-23

**Authors:** Madoka Kawano, Tomoyuki Wada, Hirofumi Anai, Takashi Shuto, Shinji Miyamoto

**Affiliations:** Department of Cardiovascular Surgery, Oita University, 1-1-1 Idai-ga-oka, Hazama, Yufu City, Oita 879-5593 Japan

**Keywords:** Ruptured coronary-pulmonary artery fistula, Coronary aneurysm, Cardiac tamponade

## Abstract

**Background:**

Coronary fistulae are occasionally detected using echocardiography or coronary angiography. We report a patient with cardiac tamponade because of a ruptured aneurysm of a coronary artery fistula.

**Case presentation:**

A 60-year-old man was referred to our hospital with sudden onset of chest pain and unconsciousness. He was initially diagnosed with cardiac tamponade for type A acute aortic dissection, and an emergency operation was performed. A large amount of bleeding was seen in the pericardium, but aortic dissection around the arch was not observed. Instead, a ruptured aneurysm of a coronary-pulmonary fistula was identified on the pulmonary artery root. The aneurysm was resected, and the fistula was closed by ligation. The patient’s postoperative progress was good, and he was discharged on postoperative day 12 without any abnormalities on the coronary arteriogram.

**Conclusions:**

Preoperative diagnosis of the rupture of the small coronary artery aneurysm is difficult in such an emergency case, and this possibility should be considered in differential diagnosis when the CT image does not show typical aortic dissection.

## Background

Coronary fistulae are occasionally detected using echocardiography or coronary angiography. In some of these cases, the fistula is accompanied by aneurysmal change, which has the possibility of rupture. The association between the size and shape of a coronary aneurysm and the risk for rupture is controversial. We report a patient with cardiac tamponade because of a ruptured aneurysm of a coronary artery fistula.

## Case presentation

A 60-year-old man with sudden onset of severe chest pain and unconsciousness was referred to another hospital. Cardiac tamponade because of Stanford type A acute aortic dissection was suspected, and the patient was transferred to our hospital. Physical examination and investigations were conducted while the patient was under tracheal intubation. He had a blood pressure of 65/40 mmHg and a pulse rate of 80/min even after an attempted pericardial drainage. Heart sounds were normal, with no murmurs or bruits. Significant laboratory findings were as follows: white blood cell count, 24,960 cells/mL; hemoglobin, 11.9 g/dL; platelet count, 185,000 cells/mL; aspartate aminotransferase, 386.5 U/L; alanine aminotransferase, 155.0 U/L; urea nitrogen, 22.77 mg/dL; creatinine, 1.56 mg/dL; and C-reactive protein, 1.65 mg/dL. Laboratory results included increased leukocytes and liver and kidney dysfunction. There was no coagulation system disorder.

**Fig. 1:**
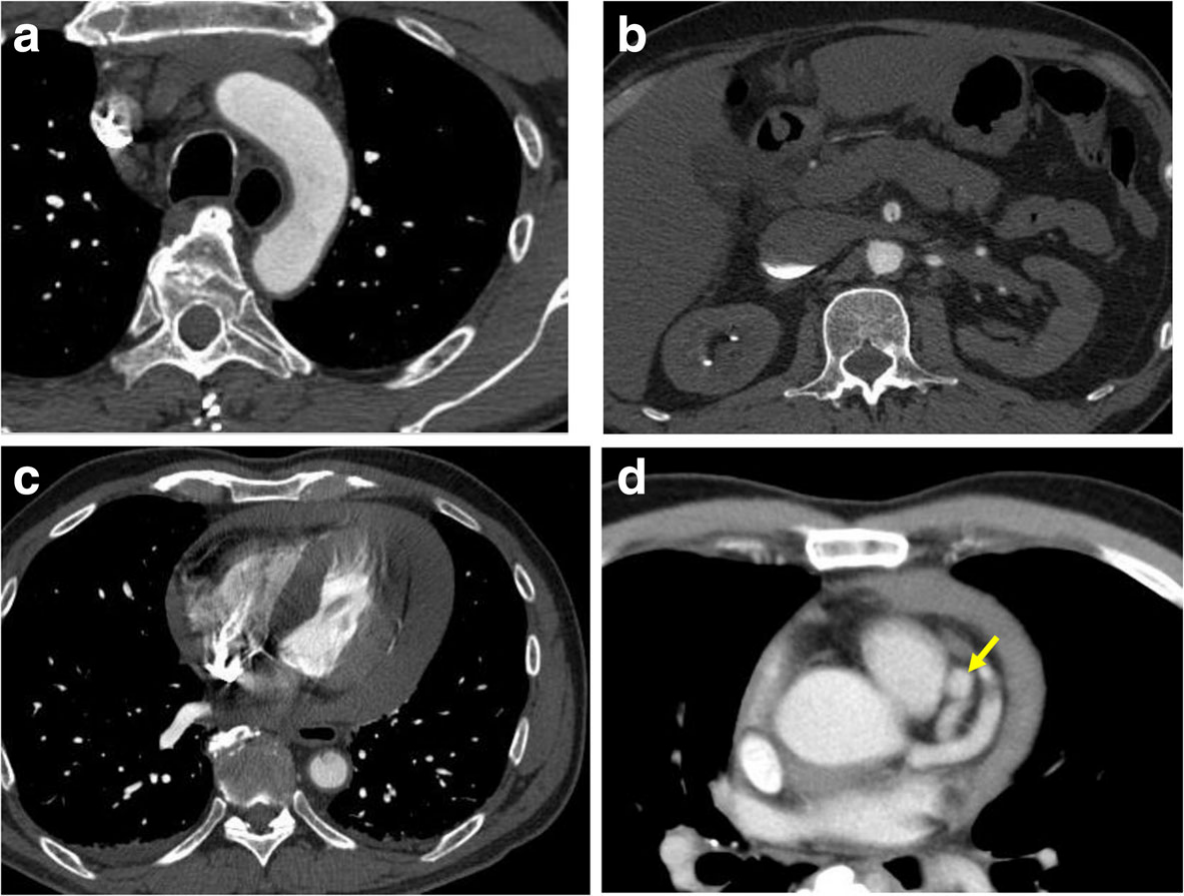
Preoperative images. Contrast-enhanced CT does not show a typical aortic dissection (**a**) and shows a dissection of the superior mesenteric artery (**b**). Cardiac tamponade was also revealed. **c** Coronary aneurysms appeared in a delayed phase (**d**)

Chest X-ray revealed mild cardiomegaly. Electrocardiography showed a normal sinus rhythm without any ischemic changes. Contrast-enhanced CT did not show typical aortic dissection (Fig. 1a) but revealed a localized dissection of the superior mesenteric artery (Fig. 1b) and cardiac tamponade (Fig. 1c).

**Fig. 2:**
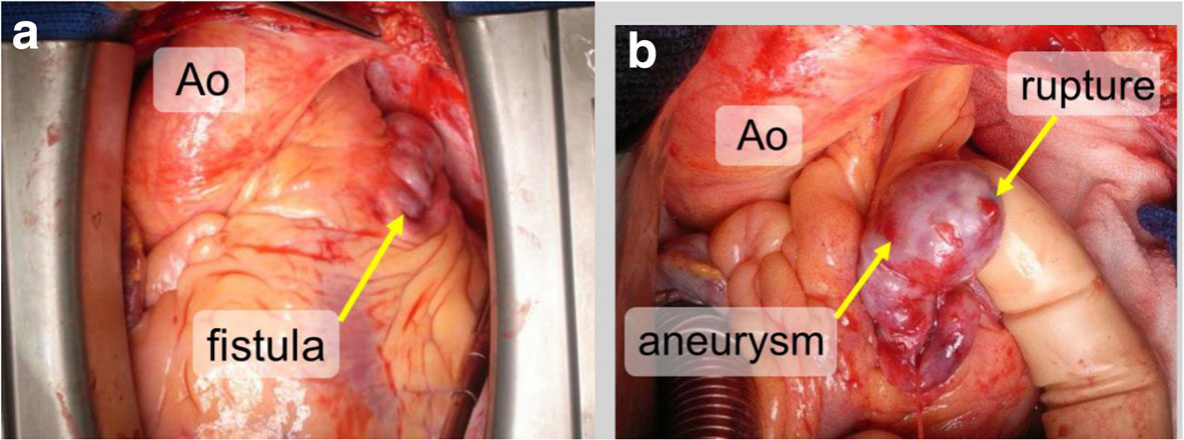
Macroscopic findings. The intraoperative macroscopic findings revealed coronary artery fistulae in front of the left atrial appendage and on the pulmonary artery root, accompanied by a 15-mm saccular-type coronary artery aneurysm (**a**). The top of the aneurysm had a papillary protrusion that ruptured (**b**)

The patient underwent emergency surgery for cardiac tamponade of unknown causes. Intraoperatively, there was a huge hematoma in the pericardium, but aortic dissection was not macroscopically observed on the aorta. Direct transaortic and transesophageal echocardiography revealed a normal ascending aorta and aortic arch. However, there were coronary artery fistulae in front of the left atrial appendage and on the root of the pulmonary artery, accompanied by a 15-mm saccular-type coronary artery aneurysm. The top of the aneurysm had a papillary protrusion that ruptured (Fig. 2). On the basis of these findings, the patient was diagnosed with a ruptured aneurysm of coronary-pulmonary fistulae. The feeder artery of the aneurysm was unknown because we did not preoperatively examine the coronary angiogram; therefore, we carefully operated.

**Fig. 3:**
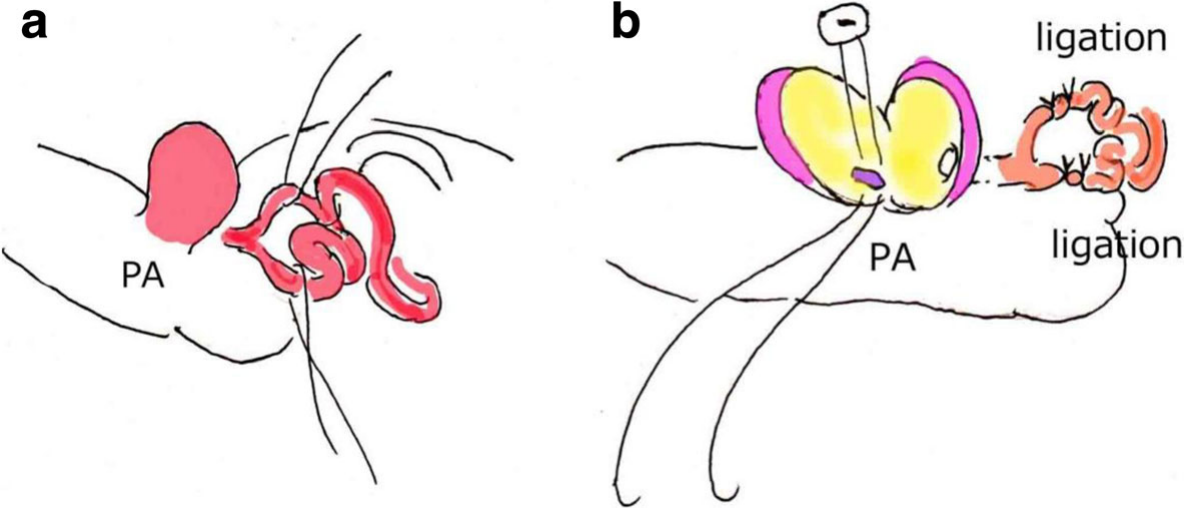
Intraoperative findings. The intraoperative findings revealed two feeder arteries and a drainage vessel. Two feeder arteries were ligated (**a**), and the last drainage vessel was closed by suturing from inside the aneurysm (**b**)

**Fig. 4:**
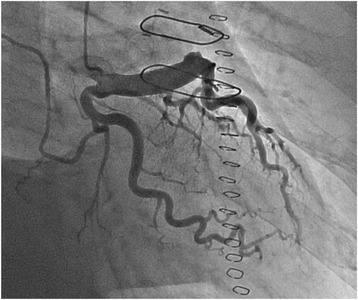
Postoperative images. Coronary angiography revealed significant ectasia from the left main trunk to the left anterior descending coronary artery. However, there was no aneurysm or fistula

We started cardiopulmonary bypass and performed on pump beating. We detected three blood vessels, two of which were feeder arteries that probably originated from the left anterior descending branch of the coronary artery and one was a drainage vessel that directly flowed to the pulmonary artery. We clamped each of these vessels and confirmed not only the electrocardiography but also that the cardiac contraction did not change. Thereafter, the aneurysm was resected, and two feeder arteries were ligated. The last drainage vessel was closed by suture from inside the aneurysm (Fig. 3). Weaning from cardiopulmonary bypass was smoothly accomplished. After surgery, electrocardiography did not reveal any ischemic changes. Coronary angiography revealed significant ectasia from the left main trunk to the left anterior descending coronary artery; however, there was no aneurysm or fistula (Fig. 4). The right coronary artery was intact. In fact, when we reviewed the preoperative CT, we understood that aneurysms appeared in a delayed phase (Fig. 1d). The patient had an uneventful recovery and was discharged on postoperative day 12.

## Discussion

It is reported that 19–26% of coronary-pulmonary artery fistulae are complicated with saccular aneurysms [1]. The causes of the aneurysmal change are attributed to arteriosclerosis, arteritis, connective tissue disorder, infection, metastatic malignancy, congenital defects, trauma, and high flow through the shunt.

Most fistulae are asymptomatic, but some cases with high shunt volume present with symptoms such as heart failure, angina, or infective endocarditis [2]. Fatal rupture of saccular aneurysms can occur. Fistulae are treated by observation, coil embolization, and surgery. Asymptomatic cases with small-sized fistulae are often just observed. Performing coil embolization has been recently more common with the improvement of catheterization techniques. However, there are still few reports of embolization for fistulae with aneurysms. The angioarchitecture is too complicated for catheterization in such cases, and there is some risk for the rupture of the embolism by the procedure.

In general, active treatment is recommended if the size of the aneurysm is > 30 mm because of the high risk for rupture [3]. However, some reports say that the size of the aneurysm associated with risk for rupture is not uniform because the size in some cases is < 10 mm, as in our case [4]. Furthermore, in some cases, aneurysms are diagnosed after pericardial drainage or conservative treatment. In our case, the correct diagnosis was unfortunately confused with a huge hematoma in the pericardium and actual superior mesenteric artery dissection, which was the diagnosis made at another hospital. The patient with shock vital did not permit a detailed examination, coronary angiography, or electrocardiogram-gated CT. The patient was definitively diagnosed with ruptured aneurysm of a coronary-pulmonary artery fistula during the operation. It is necessary to consider not only aortic dissection or myocardial infarction but also ruptured aneurysm of a fistula as a differential diagnosis if the origin of acute cardiac tamponade with bloody pericardial effusion is not clear. Following this experience, we should consider active treatment, including surgery and follow-up, even if we find only asymptomatic or small aneurysms.

When surgery was previously performed for coronary-pulmonary artery fistulae, we ligated the fistulae without using cardiopulmonary bypass. In recent years, safe and stable cardiopulmonary bypass surgery has become established, and we should use cardiopulmonary bypass in the case of multiple fistulae because we can prevent fistula remnants by directly closing the fistula from the pulmonary artery [5]. In addition, if the preoperative examination and diagnosis are not sufficient like our case, we consider that the on-pump method is effective because we can confirm shunt bleeding from the fistula orifice and perfectly close the fistula.

Because we were able to see the orifices of the fistulae in this case, we directly closed them and could resect the aneurysm, and we obtained good results. Fortunately, we can prevent residual fistulae, but it is difficult to identify all abnormal vessels and ligate those that have complicated angioarchitecture.

## Conclusions

We report a rare case of a rupture of an aneurysm of a coronary-pulmonary fistula that was difficult to correctly diagnose before performing emergency surgery.
